# Design and Preparation of Polyimide/TiO_2_@MoS_2_ Nanofibers by Hydrothermal Synthesis and Their Photocatalytic Performance

**DOI:** 10.3390/polym14163230

**Published:** 2022-08-09

**Authors:** Zhenjun Chang, Xiaoling Sun, Zhengzheng Liao, Qiang Liu, Jie Han

**Affiliations:** 1 College of Materials Science and Engineering, Jiangsu University of Science and Technology, Zhenjiang 212003, China; 2 Polytex Engineering Group, Yangzhou 225000, China

**Keywords:** nanofibers_1_, TiO_2_, MoS_2_, polyimide

## Abstract

Organic–inorganic nanocomposite fibers can avoid the agglomeration of single nanoparticles and reduce the cost (nanoparticles assembled on the surface of nanofibers), but also can produce new chemical, electrical, optical, and other properties, with a composite synergistic effect. Aromatic polyimide (PI) is a high-performance polymer with a rigid heterocyclic imide ring and an aromatic benzene ring in its macromolecular framework. Due to its excellent mechanical properties, thermal stability, and easy-to-adjust molecular structure, PI has been widely used in electronics, aerospace, automotive, and other industries related to many applications. Here, we report that TiO_2_ nanorods were grown on polyimide nanofibers by hydrothermal reaction, and MoS_2_ nanosheets were grown on TiO_2_ nanorods the same way. Based on theoretical analysis and experimental findings, the possible growth mechanism was determined in detail. Further experiments showed that MoS_2_ nanosheets were uniformly coated on the surface of TiO_2_ nanorods. The TiO_2_ nanorods have photocatalytic activity in the ultraviolet region, but the bandgap of organic/inorganic layered nanocomposites can redshift to visible light and improve their photocatalytic performance.

## 1. Introduction

With the increases in social development and people’s living standards, environmental pollution has received more and more attention. Indoor and outdoor air, water, and soil pollution have seriously affected people’s health and normal lives [1,2]. Using the photocatalytic properties of semiconductor materials, organic pollutants in water or air can be completely degraded into carbon dioxide and water. This method has been widely used for the treatment of wastewater and gas, and has a significant advantage over the electrocatalytic and wet catalytic techniques requiring high temperature for the decomposition of refractory toxic organic compounds [3,4,5]. When the catalyst has a nanostructure, it produces significant surface and size effects. Nanocatalysts have a huge surface area relative to the general size of the catalyst, which expands the surface of the atomic number. With the reduction in size, the pore size channel becomes very short with a large number of edges and steps, which increases the surface activity of the catalyst. However, it easily agglomerates, thereby affecting its dispersibility and utilization, and it is necessary to use carrier materials as a template for uniform dispersion. The electrospun fibers have a small fiber diameter, good flexibility, and ease of operation. Catalytic carriers can produce a strong synergistic effect in the catalyst, which increases the catalytic performance. In addition, some Pt, Au, and other precious metals are expensive as catalysts. The use of electrospun fiber as a template of catalyst can effectively overcome the above shortcomings [6]. Studies showed that the metal oxides made of nanoparticles have an increased quantum size effect, the energy levels of the conduction band and valence band are separate, and they can be widened with a higher redox capacity. As the particle size decreases, the combination of photogenerated electrons and holes reduces, the time of photo-generated electrons from the crystal shortens, and the electron-to-hole separation effect improves, thus increasing the photocatalytic efficiency. Additionally, the particle size of the semiconductor catalyst is reduced, the surface area is increased, the ability of the adsorbed substrate is enhanced, and the photocatalytic reaction can be promoted. Therefore, metal-oxide nanoparticles with stronger catalytic ability can be supported on the electrospinning fibers to obtain a new type of photocatalytic material, which is superior to traditional catalysts in terms of catalytic efficiency and operability [7,8]. Titanium dioxide (TiO_2_) is a representative material used for semiconductor photocatalysts for environmental purification applications [9,10] and hydrogen generation [11,12,13]. Although TiO_2_ has a strong oxidizing property, high strength, and good chemical stability, making it suitable as a photocatalyst, the main drawback is its wide band gap of 3.23 eV. This wide band gap means that it is only effective in the ultraviolet region of the solar spectrum, which accounts for only 5% of the total incident solar energy [14,15,16,17,18]. This can be remedied by sensitizing it with a narrow bandgap semiconductor or by forming a new donor state below the TiO_2_ conduction band.

At present, 2D semiconductor materials have recently attracted attention, mainly due to the excellent electrical properties of the two-dimensional structure of the thin layer of atoms. Among the various inorganic 2D layered materials, molybdenum disulfide (MoS_2_) has been studied by many researchers due to its good electrical, mechanical, optical, magnetic, and electrochemical properties. Furthermore, MoS_2_ nanosheets/nanomaterials can be used in many applications in diverse fields, such as energy storage, gas-sensing, catalyst, and microwave absorbers [19]. The inherent MoS_2_ is an n-type semiconductor with a band gap in the range of 1.2–1.9 eV, as determined by the number of layers [20,21]. Recently, theoretical and experimental studies have shown that MoS_2_ nanosheets can be used as a good catalyst for the decomposition and degradation of pollutants by coupling with semiconductor photocatalysts, such as CdS [22,23,24], C_3_N_4_ [25,26], ZnO [27], Bi_2_WO_6_ [28] and ZnIn_2_S_4_ [29]. Therefore, it is thought that the combination of two-dimensional MoS_2_ nanosheets and one-dimensional titanium dioxide to form three-dimensional heterostructures can effectively utilize the synthesis advantages of TiO_2_ nanorods and MoS_2_ nanosheets to form a photocatalyst with excellent properties. We expect the MoS_2_ nanosheets used in heterostructures not only to promote charge separation, but also to provide more active sites for photocatalytic reactions.

Electrospun polyimide (PI) nanofibers can be used as the skeleton of nanocatalysts. Some nanoparticles/nanorods can be decorated on the surface of nanofibers by physical/chemical interactions (e.g., hydrogen bonds, electrostatic forces, and other interactions between functional groups) [30,31,32]. In this study, an electrospinning technology and hydrothermal method were used to develop a simple and effective strategy to prepare highly dispersed TiO_2_ nanorods with good growth on electrospun PI nanofibers. The MoS_2_ nanoparticles were grown on the surface of the TiO_2_ nanorods by a hydrothermal method to form nanocomposite fibers with heterostructure. This provided a high-level hybrid structure for the manufacture of functionalized nanofibers for multiple purposes.

## 2. Experimental Section

### 2.1. Materials

Pyromellitic dianhydride (PMDA) and 4,4′-oxydianiline (4,4’-ODA) were of chemical reagent grade and purchased from Sinopharm Chemical Reagent Co. Ltd. (Shanghai, China), and they were further purified by sublimation before use. Titania powder, hydrochloric acid (HCl), sodium molybdate (Na_2_MoO_4_·2H_2_O), thioacetamide (CH_3_CSNH_2_), N,N-dimethylformamide(DMF), and anhydrous ethanol were purchased from Sinopharm Chemical Reagent Shanghai Co., Ltd.

### 2.2. Preparation of Polyimide

**Figure 1:**
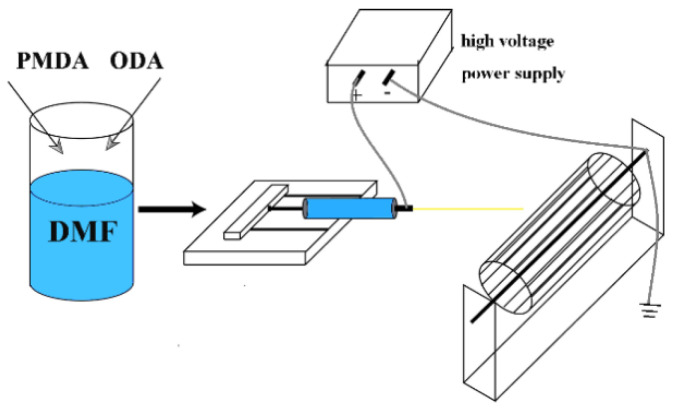
Synthesis of polyamic acid (PAA) solution and the process of electrospinning.

In this experiment, polyimide nanofibers were prepared by a two-step method. Both 3.97 g ODA and 4.33 g PMDA were reacted in 35 mL of N,N-dimethylformamide (DMF) by polycondensation, with a solid content of 25%. The solution became highly viscous and we stopped stirring after 8 h. The solution of the precursor of polyamide acid (PAA) was obtained. The PAA nanofibers were prepared by electrospinning the PAA solution (15%) at 15 kV with 15 cm from needle to collector. After electrospinning (KD Scientific 100 and Tianjin Dongwen 30KVDC) the PAA solution, we obtained the electrospun polyamide acid nanofibers, then polyamide acid nanofibers proceeded with thermal imidation. Finally, polyimide nanofibers were prepared. Figure 1 illustrates the electrospinning process in the present work.

### 2.3. Synthesis of the PI/TiO_2_ Nanofibers

In the hydrothermal reaction autoclave, 33 mL water solution with 1 mol/L hydrochloric acid was added, then 0.04~0.05 g of titanium powders was added; finally, the polyimide fibers (0.1 g) were added. We then sealed it and set it aside for 3 h. The reaction kettle was put into the oven, heated to 160 °C, and reacted for 16 h. After the reaction, we removed the product, washed it with deionized water, and dried it at 60 °C.

### 2.4. Synthesis of the PI/TiO_2_@MoS_2_ Nanofibers

A small amount of sodium molybdate and thioacetamide was added to the deionized water, and the solution was stirred and put into to the hydrothermal reaction autoclave. The PI/TiO_2_ fibers were then added into the autoclave reactor; the autoclave reactor was sealed and put it in the oven heated to 200 °C and left to react for 24 h. Then, the product of the reaction was washed with deionized water several times and put in the oven at 60 °C to dry.

### 2.5. Characterization

The morphologies of the as-obtained samples were observed using field-emission scanning electron microscopy (FE-SEM, Carl Zeiss Merlin Compact and Hitachi S4800). The crystal structure of the products was characterized by X-ray diffraction (XRD, Bruker D8 Advance diffractometer) using CuKa1 radiation (λ = 0.15406 nm). UV–Vis absorption spectra were obtained using a UV–Vis spectrophotometer (UV-3600, Shimadzu, Japan). FTIR spectra were recorded on a Varian 670-IR spectrometer. The UV lamp was from Shanghai Guanghao ZF-2 (365 nm). The UV–Vis diffuse reflectance spectra (DRS) were recorded using UV–V is spectrophotometer (V-650.Jasco). X-ray photoelectron spectroscopy (XPS) measurements were carried out with an ESCALAB 250 Xi photoelectron spectrometer using Al Ka radiation (ThermoFisher Scientific, Waltham, MA, USA).

## 3. Results and Discussion

### 3.1. Characterization of the As-Prepared Photocatalysts

**Figure 2:**
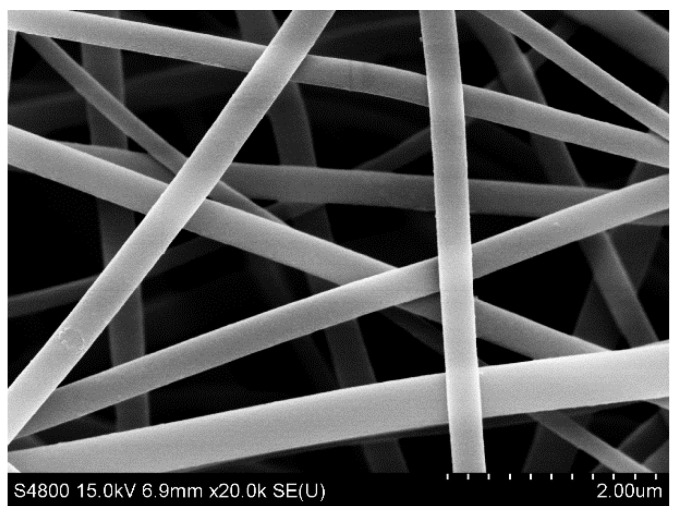
FESEM images of PI nanofibers.

**Figure 3:**
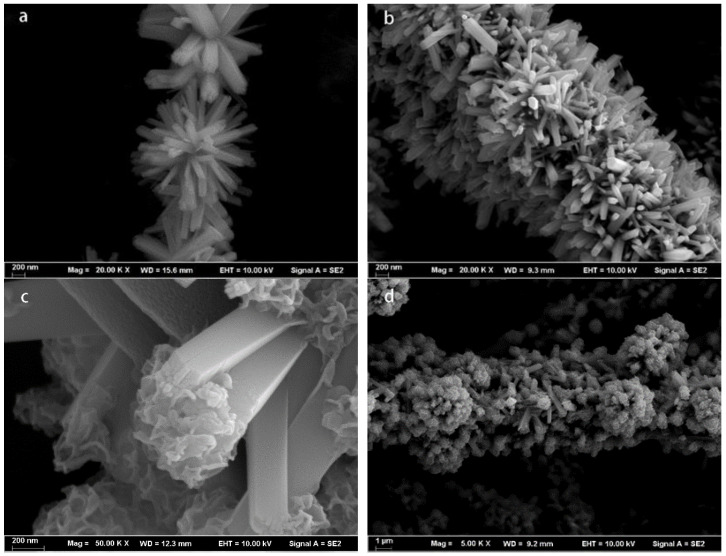
FESEM images of PI/TiO_2_ nanocomposites (**a**,**b**) and PI/TiO_2_@MoS_2_ nanocomposites (**c**,**d**).

The morphology and nanostructure of the PI nanofibers were characterized by field-emission scanning electron microscopy (FESEM) observations (Figure 2). The surface morphology of the rod-like TiO_2_ with a length ranging from 800 to 1000 nm and a width ranging from 50 to 200 nm could be clearly observed, as shown in Figure 3a,b. From the figure, we can see that the TiO_2_ nanorods were very dense in the distribution of polyimide fibers. Figure 3c,d show that MoS_2_ nanospheres were grown in situ on the surface of the TiO_2_ nanorods. We can see that the MoS_2_ nanospheres were evenly distributed on the surface of TiO_2_ nanorods, which notably increased the specific surface area.

**Figure 4:**
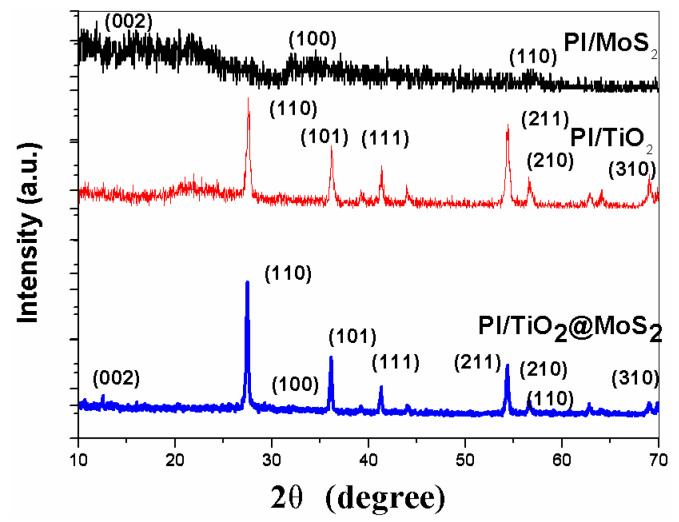
XRD pattern of the as-prepared PI/MoS_2_, PI/TiO_2_ and PI/TiO_2_@ MoS_2_. 2θ.

The PI/TiO_2_@MoS_2_ nanocomposite was characterized by powder X-ray diffraction (XRD), as shown in Figure 4. Strong XRD diffraction peaks at 2θ = 27.45°, 36.09°, 41.23°, 54.32°, 56.64°, and 69.01° were clearly observed, consistent with the (110), (101), (111), (211), (220), and (301) faces, respectively, of rutile phase TiO_2_ indexed to the JCPDS card 21-1276 with a space group of P42/mnm (a = b = 4.593Å and c = 2.959Å), similar to the standard spectrum of rutile TiO_2_. The hexagonal phase MoS_2_ was clearly shown. The diffraction weak peaks could be assigned to the (002), (100), and (110) planes in the hexagonal phase MoS_2_ (a = b = 0.316 nm, c = 1.230 nm, JCPDS card no. 37-1492), consistent with the findings of previous studies [33].

**Figure 5:**
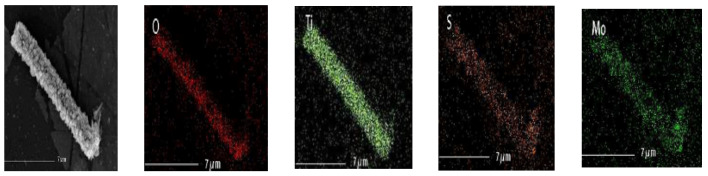
EDS mapping results from PI/TiO_2_@MoS_2_.

To further confirm the surface layer was MoS_2_, energy dispersive X-ray spectrometry (EDS) mapping (Figure 5) analysis of the PI/TiO_2_@ MoS_2_ nanocomposites was conducted. The EDS pattern of the TiO_2_@MoS_2_ nanorods heterostructures showed that the product nanorods were composed of Ti, O, Mo, and S. The EDS elemental mapping supports our argument that the outer layer was MoS_2_ nanosheets and the inner layer was TiO_2_ nanorods. The above experimental results proved that only a few layers of MoS_2_ were wrapped on the surface of the ultrafine TiO_2_ nanobelts to form TiO_2_@MoS_2_ nanorod heterostructures.

**Figure 6:**
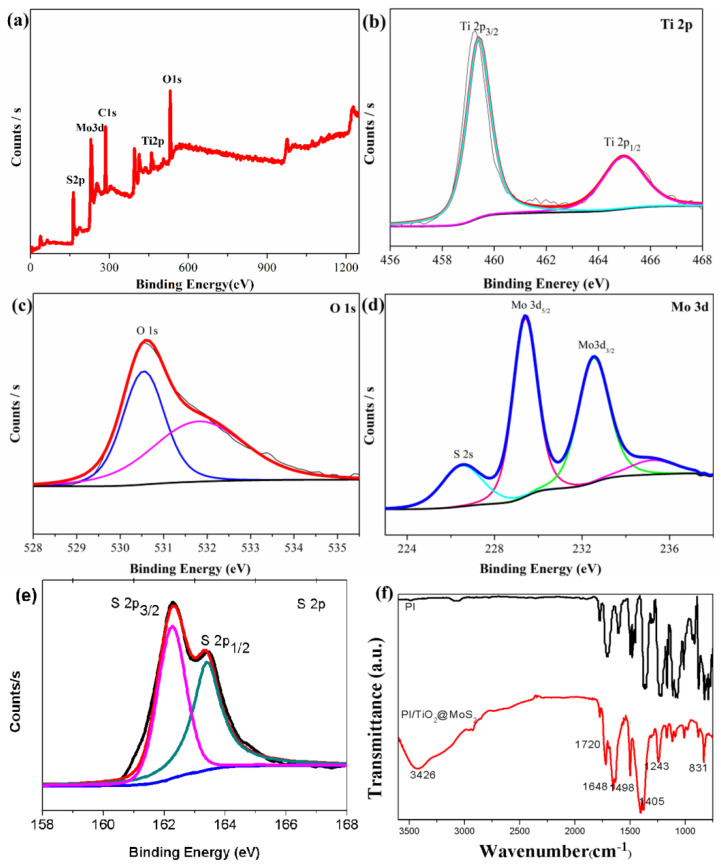
(**a**) XPS spectrum of PI/TiO_2_@MoS_2_ composite; (**b**–**e**) high-resolution XPS spectra of Ti2p, O1s, Mo3d, and S2p; (**f**) FT-IR spectra of the nanocomposite.

The chemical composition and valence state were characterized by X-ray photoelectron spectroscopy (XPS). The full-range XPS spectra of PI/TiO_2_@MoS_2_ (0–1050 eV) are shown in Figure 6a. Figure 6b shows that the binding energies (BE) of Ti 2p_3/2_ and Ti 2p_1/2_ were 459.3 and 464.7 eV, respectively, which we ascribed to the Ti^4+^ oxidation state. In Figure 6c, O1s is shown, and it is useful for identifying the core levels. In Figure 6d, the high-resolution XPS spectra show that the binding energy of Mo 3d_3/2_ and Mo 3d_5/2_ peaks in the TiO_2_@MoS_2_ heterostructures located at 229.5 and 232.8 eV, respectively, indicating that the Mo element was present in the Mo^4+^ chemical state. Figure 6e shows that the binding energies of S 2p_3/2_ and S 2p_1/2_ were 162.3 and 163.8 eV, respectively. By comparison to the NIST X-ray Photoelectron Spectroscopy Database, we identified the S element corresponding to producing the material for MoS_2_.

PAA was analyzed in our previous report [34]. The FTIR of PI is shown without the –OH peak in Figure 6f. The PI/TiO_2_ nanorods showed bands around 3426 and 1648 cm^−1^, corresponding to the stretching and bending vibrations of hydroxyl groups on the surface of the TiO_2_ nanorod surface, respectively (Figure 6f). The strong absorption band between 800 and 400 cm^−1^ was attributed to the Ti-O and Ti-O-Ti vibrations. The strong characteristic absorption peaks at 3426, 1720, 1648, and 1498 cm^−1^ in Figure 6f also indicated the presence of amide groups, while the absorption peak at 1648 cm^−1^ represented the tertiary amide, which indicated that imidization was completed. The peaks at 1405 and 725 cm^−1^ corresponded to the asymmetric stretching vibration of C–N and the deformation of the imide ring, respectively, which create the characteristic peaks of polyimide groups. The bands near 1648 and 1117 cm^−1^ were also assigned to the Ti-O and Ti-O-C stretching modes, respectively. The FTIR results indicated that the TiO_2_ nanoparticles were successfully coated on the polyimide matrix. In MoS_2_, FTIR peaks exist in the range from 1008 to 1648, 2925, and 3426 cm^−1^. The strong O-H peak and water bonding are indicated by 3426 and 603 cm^−1^, respectively. The peaks situated at 1008 and 1243 cm^−1^ occurred due to the formation of complex sulfur with the active sites in MoS_2_.

**Figure 7:**
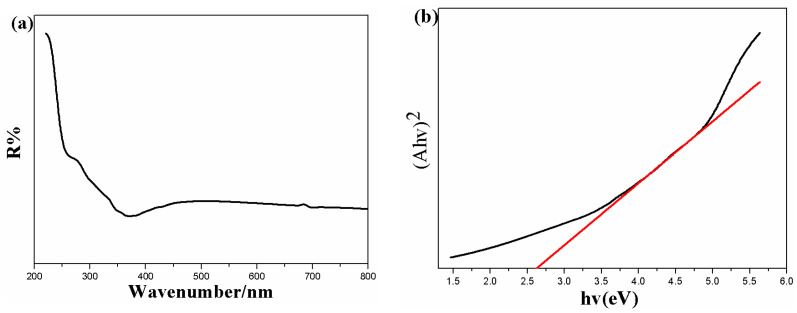
(**a**) UV–Vis DRS absorbance spectra and (**b**) plot of (Ahν)^2^ versus hν for the samples.

To evaluate the effects of MoS_2_ nanoparticles onto the TiO_2_ nanorod support on the optical properties of PI/TiO_2_@MoS_2_ nanocomposite, UV–Vis diffuse absorbance spectra (DRS) analysis was performed. Figure 7a shows the corresponding UV–Vis DRS spectra for the PI/TiO_2_@MoS_2_ samples. The absorption thresholds for the samples were obtained from the UV–Vis DRS curves by extrapolating the tangent lies of the spectra. In general, bare TiO_2_ nanoparticles show absorption in the UV region without any absorption in the visible range owing to its wide band gap (~3.2 eV). As shown in Figure 7a, the threshold wavelength for the synthesized PI/TiO_2_@MoS_2_ sample is about 450 nm. Compared with pure TiO_2_, a red shift in the absorption edges toward the visible region was observed in the nanocomposite. This was induced by the strong optical absorption of black MoS_2_ in the visible-light region, which illustrated that there were interactions between TiO_2_ and MoS_2_ on the interface. The band gap energies (Eg) of the samples were calculated using the equation (Ahν)^2^ = K(hν−Eg), where hν is the energy of a photon (eV), A is the absorption coefficient, K is a constant, and Eg is the band gap. The band gap was calculated by extrapolating the linear part of the spectra in a diagram of (Ahν)^2^ versus the photon energy (Figure 7b). The band gap energy value for the PI/TiO_2_@MoS_2_ nanocomposite was calculated as 2.7 eV, implying that MoS_2_ nanospheres on a TiO_2_ nanorod support decreased the optical band gap energy. Therefore, the electron–hole separation was relatively better in the PI/TiO_2_@MoS_2_ nanocomposite.

### 3.2. Photocatalytic Activity the As-Prepared Photocatalysts

**Figure 8:**
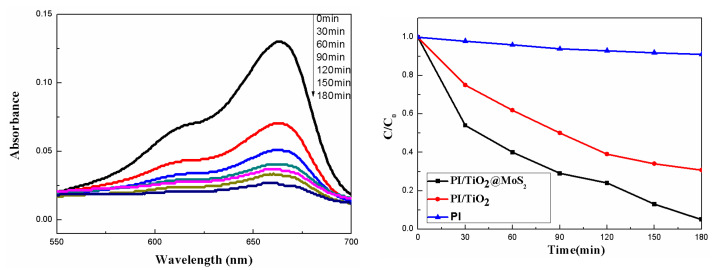
UV–Vis absorption spectra of methylene blue (MB) as a function of UV-light irradiation time (λ = 365 nm) in the presence of the PI/TiO_2_@MoS_2_ composites fiber (left); MB concentration changes with the as-prepared samples (right).

The photocatalytic performance of PI/TiO_2_@MoS_2_ was evaluated for the photodegradation of methylene blue (MB) at room temperature. The decay of the characteristic absorption peak of MB at 663 nm was followed every 30 min by UV–Vis spectrophotometry. Before UV exposure, the solution was kept in the dark for 30 min to build the adsorption/desorption equilibrium between the dye and surface. We found a gradual decrease in the main absorption peak, because of the adsorption of MB molecules on the surface of the sample. Usually, it is difficult to degrade pure MB and PI nanofiber in UV [35,36]. Figure 8 (left) shows the UV–Vis spectra of MB (5 mg/L) after ultraviolet light (λ = 365 nm) irradiation in the presence of PI/TiO_2_@MoS_2_ (0.02 g). With increasing irradiation time, the intensity of the characteristic absorption band of MB at 663 nm markedly reduced. In the meantime, the color of the solution changed, turning from blue to colorless after 180 min with irradiation, thus indicating the gradual decomposition of MB molecules during ultraviolet-light irradiation. The degradation efficiency is reported as C/C_0_, where C is the absorption of the main peak at 663 nm of MB at time t, and C_0_ corresponds to the initial concentration (after achievement of adsorption/desorption equilibrium (30 min)). As shown in Figure 8 (right), we observed that the dye degradation rate of the prepared samples varied with the same irradiation rate of UV at the same time. The results showed that PI/TiO_2_@MoS_2_ composites showed enhanced photocatalytic activity compared with PI/TiO_2_ for the degradation of methylene blue under UV irradiation. The PI/TiO_2_@MoS_2_ composites decomposed about 95% methylene blue within 180 min under UV irradiation. Compared with a single PI/TiO_2_ composite fiber or PAN nanofibers with MoS_2_-TiO_2_ surface-loaded by vacuum filtration [37], a certain amount of molybdenum dioxide increased the efficiency of photodegradation. It may promote the light absorption efficiency of TiO_2_ particles that decreases the electron–hole recombination and enhances the photogenerated charge separation.

### 3.3. Possible Mechanism of the Experiment

**Figure 9:**
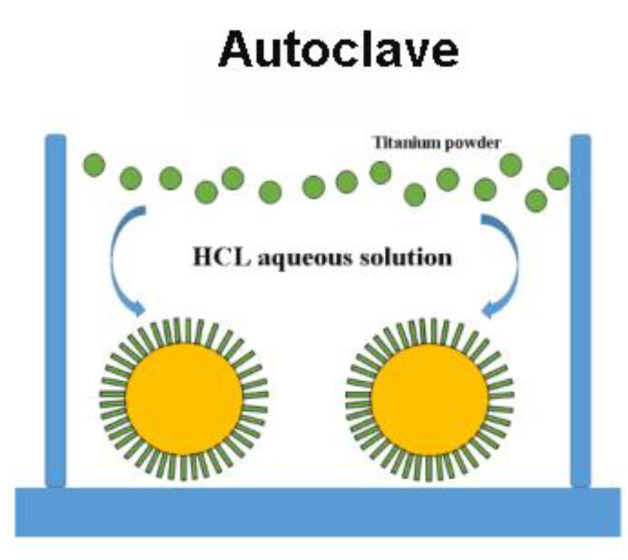
Schematic diagram of the formation mechanism of TiO_2_ nanorods.

Figure 9 shows a schematic diagram of the formation mechanism of TiO_2_ nanorods by the “dissolve and grow” progress, which we describe using the following chemical reaction:

$$2\mathrm{Ti}+6\mathrm{HCl}\to 2{\mathrm{TiCl}}_{3}+3H_{2}\left(g\right)$$

$${\mathrm{Ti}}^{3+}+H_{2}O\to {\mathrm{TiOH}}^{2+}+H^{+}$$

$${\mathrm{TiOH}}^{2+}+O_{2}^{-}\to \mathrm{Ti}\left(\mathrm{IV}\right)-\mathrm{oxo}\;\mathrm{species}+O_{2}^{-}\to {\mathrm{TiO}}_{2}$$

At the beginning, Ti powders react with H^+^ at high temperature and gradually dissolve in the solution of HCL, continuously releasing the Ti(III) precursors into the reaction solution. Due to the instability of Ti(III) in aqueous solution, Ti(III) is hydrolyzed to TiOH^2+^. According to Fujihara et al. [38], TiOH^2+^ is oxidized to Ti(IV) by reacting with dissolved oxygen. Therefore, the formation mechanism of rutile TiO_2_ nanorods using Ti(IV) complex ions as growth units can be described as follows: For rutile TiO_2_, TiO_6_ octahedra are first formed by bonding Ti atoms and six oxygen atoms. Then, the TiO_2_ octahedron shares a couple of opposite edges with the next octahedron, forming a catenarian structure. The growth of rutile nanorods follows the sequence (110) < (100) < (101) < (001), because the growth rate of different crystal planes depends on the number of coordinated polygon body corners and edges. Therefore, rutile TiO_2_ nanorods grown in the [001] direction were formed [39,40].

**Figure 10:**
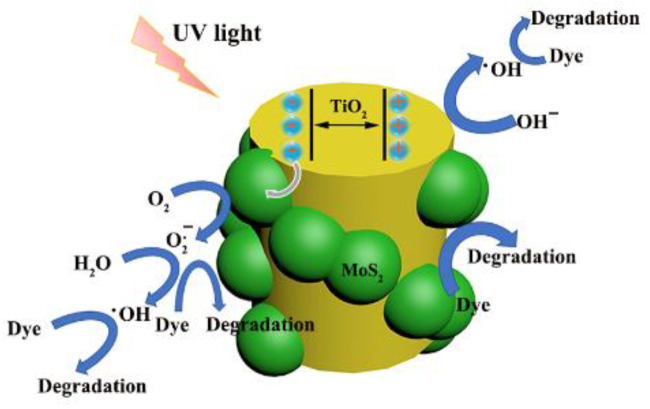
Assumed mechanism of the photodegradation of dyes with PI/TiO_2_@MoS_2_ heterostructures.

According to the previous experimental results, a promotional mechanism is shown in Figure 10. Under UV-light irradiation, TiO_2_ absorbs photons and creates electron–hole pairs. Because the conduction band (CB) of TiO_2_ is higher than that of MoS_2_ [41,42,43], the photoelectrons generated by CB of TiO_2_ are easily transferred to MoS_2_, which improves the separation of photogenerated electron–hole pairs and enhances the photocatalytic activity of PI/TiO_2_@MoS_2_ heterostructures. The separated electrons react with dissolved O_2_ to produce O_2_^.−^ radicals on the surface of MoS_2_ nanosheets. Next, they combine with H**^+^** to produce H_2_O_2_, and finally decompose into ·OH. In the meantime, the cumulative holes on the surface of TiO_2_ are trapped by OH^–^ or H_2_O to form hydroxyl radicals, ·OH. Finally, the oxidation of organic dyes mainly occurs due to the involvement of holes, ·OH, and O_2_^.−^ radicals. The main reactions in our study are described by equations:

$${\mathrm{TiO}}_{2}+\mathrm{hv}\to {\mathrm{TiO}}_{2}\left(e^{-}+h^{+}\right)$$

$${\mathrm{TiO}}_{2}\left(e^{-}+h^{+}\right)+{\mathrm{MoS}}_{2}\to {\mathrm{MoS}}_{2}\left(e^{-}\right)+{\mathrm{TiO}}_{2}\left(h^{+}\right)$$

$$e^{-}+O_{2}\to O^{.}{}_{2}^{-}$$

$$O_{2}^{\cdot -}+H_{2}O\to {\mathrm{HO}}_{2}^{\cdot }+{\mathrm{OH}}^{-}$$

$${\mathrm{HO}}_{2}^{\cdot }+H_{2}O\to H_{2}O_{2}+\cdot \mathrm{OH}$$

$$H_{2}O_{2}\to 2\cdot \mathrm{OH}$$

$$h^{+}+{\mathrm{OH}}^{-}\to \cdot \mathrm{OH}$$

$$\cdot \mathrm{OH}/O^{.}{}_{2}^{-}/h^{+}+\mathrm{dye}\to {\mathrm{CO}}_{2}+H_{2}O\;$$

## 4. Conclusions

PI/TiO_2_@MoS_2_ heterostructures were successfully fabricated by the assembly of MoS_2_ nanosheets and TiO_2_ nanorods on electrospun polyimide nanofibers using a simple hydrothermal method. Our innovation is in the successful structure of nanofiber–nanorod–nanosheet multilevel nanostructure of PI/TiO_2_@MoS_2_ composite fibers. Compared with the usual nanoparticles on the surface of electrospun nanofibers, the functionalized application of composite nanofibers is expected. In this study, nanocomposite fibers with a multistage structure were proposed, which improves the performance of a single composite and can realize various functional applications. This multistage structure not only improves photocatalytic performance, but also the choice of gas adsorption, gas separation, supercapacitors, bi-sensing, etc. These nanocomposite fibers will have a wide range of applications, which is our next research direction.

## Data Availability

The data presented in this study are available on request from the corresponding author.
